# An Almond-Based Low Carbohydrate Diet Improves Depression and Glycometabolism in Patients with Type 2 Diabetes through Modulating Gut Microbiota and GLP-1: A Randomized Controlled Trial

**DOI:** 10.3390/nu12103036

**Published:** 2020-10-03

**Authors:** Mengxiao Ren, Huaiyu Zhang, Jindan Qi, Anni Hu, Qing Jiang, Yunying Hou, Qianqian Feng, Omorogieva Ojo, Xiaohua Wang

**Affiliations:** 1 School of Nursing, Medical College, Soochow University, Suzhou 215006, China; 20185231008@stu.suda.edu.cn (M.R.); 20184231002@stu.suda.edu.cn (J.Q.); 20174231001@stu.suda.edu.cn (A.H.); jiangqing2015@suda.edu.cn (Q.J.); houyunying@suda.edu.cn (Y.H.); 20195231027@stu.suda.edu.cn (Q.F.); 2 School of Medicine, University of California, San Francisco, CA 94110, USA; huaiyu.zhang@ucsf.edu; 3 School of Health Sciences, Faculty of Education, Health and Human Sciences, University of Greenwich, London SE9 2UG, UK; o.ojo@greenwich.ac.uk

**Keywords:** type 2 diabetes, diet, carbohydrate, almond, depression, HbA1c, microbiota, short-chain fatty acids, GLP-1

## Abstract

Background: Alow carbohydrate diet (LCD) is more beneficial for the glycometabolism in type 2 diabetes (T2DM) and may be effective in reducing depression. Almond, which is a common nut, has been shown to effectively improve hyperglycemia and depression symptoms. This study aimed to determine the effect of an almond-based LCD (a-LCD) on depression and glycometabolism, as well as gut microbiota and fasting glucagon-like peptide 1 (GLP-1) in patients with T2DM. Methods: This was a randomized controlled trial which compared an a-LCD with a low-fat diet (LFD). Forty-five participants with T2DM at a diabetes club and the Endocrine Division of the First and Second Affiliated Hospital of Soochow University between December 2018 to December 2019 completed each dietary intervention for 3 months, including 22 in the a-LCD group and 23 in the LFD group. The indicators for depression and biochemical indicators including glycosylated hemoglobin (HbA1c), gut microbiota, and GLP-1 concentration were assessed at the baseline and third month and compared between the two groups. Results: A-LCD significantly improved depression and HbA1c (*p* < 0.01). Meanwhile, a-LCD significantly increased the short chain fatty acid (SCFAs)-producing bacteria *Roseburia*, *Ruminococcus* and *Eubacterium*. The GLP-1 concentration in the a-LCD group was higher than that in the LFD group (*p* < 0.05). Conclusions: A-LCD could exert a beneficial effect on depression and glycometabolism in patients with T2DM. We speculate that the role of a-LCD in improving depression in patients with T2DM may be associated with it stimulating the growth of SCFAs-producing bacteria, increasing SCFAs production and GPR43 activation, and further maintaining GLP-1 secretion. In future studies, the SCFAs and GPR43 activation should be further examined.

## 1. Introduction

Type 2 diabetes mellitus (T2DM) is a heterogenous set of disorders characterized by glycometabolism abnormalities. According to a report of the International Diabetes Federation (IDF), there are currently around 415 million people with diabetes worldwide, a figure likely to increase to 629 million by 2045 [1]. Diabetes is associated with several complications such as mental health-related disorders, particularly depressive disorders [2]. A meta-analysis revealed that the prevalence of depression in patients with diabetes was considerably higher than in non-diabetic subjects (17.6% vs. 9.8%) [3].Furthermore, epidemiological evidence indicated that patients with T2DM are almost three times as likely to be affected by depression compared with the general population [4]. A bidirectional relationship exists between T2DM and depression; depression increases the risk of the development of T2DM and the subsequent risks of hyperglycemia, insulin resistance, and micro and macro vascular complications. Conversely, a diagnosis of T2DM increases the risk of incident depression and can contribute to a more severe course of depression [2]. The American Diabetes Association (ADA) [5] and IDF [6] emphasize that: management of mental health is an important aspect of diabetes treatment. However, the tricyclic antidepressant commonly used in clinical practice has an adverse effect on glucose homeostasis control by reducing the insulin secretory rate [7].

Medical nutrition therapy is an essential measure to prevent and control diabetes at any stage of its natural course, and it improves blood glucose by regulating the nutrient and energy intake. Nevertheless, most available nutritional therapy mainly focuses on improving glycometabolism, and little attention is paid to improving the depression in T2DM patients. In China, low-fat diet (LFD) is recommended by ***Guideline for prevention and control of type 2 diabetes in China (2017 Edition)*** to help improve blood glucose levels in patients with T2DM [8]. However, a meta-analysis which included a total of nine studies and pooled results suggested a reasonable low-carbohydrate diet (LCD) is more beneficial for T2DM [9]. LCD refers to a dietary strategy that reduces the energy supply ratio of carbohydrates and increases the energy supply ratio of fats and proteins [10]. According to the energy supply ratio of carbohydrates, it can be divided into two modes: very low LCD (<26%), LCD (26~45%) [11].

In terms of the relationship between the proportion of carbohydrate intake and depression, the results remain controversial. Most studies support a protective effect of a low fat-high carbohydrate diet on depression. Pellegrin et al. [12] showed that daily carbohydrate intake was negatively correlated with depression score (R = −0.33, *p* < 0.01) in obese women. Deijen et al. [13] found that long-term consumption of LCD would aggravate negative psychology such as depression. A randomized controlled trial (RCT) by Rosenthal et al. [14] showed that a carbohydrate-rich meal could reduce depression level in patients with seasonal affective disorder. However, for T2DM patients, carbohydrate is by far the greatest dietary contributor to blood glucose rise, as expected, dietary carbohydrate restriction reliably reduces glucose profile [9]. So, it is crucial to explore a diet regimen that is beneficial to both depression and glycometabolism.

Nuts as part of LCD have been shown to effectively improve glycosylated hemoglobin (HbA1c) levels and fasting blood glucoses (FBG) in T2DM patients [15]. In addition, the synergy and interaction of all the nutrients and other bioactive components in nuts have a beneficial effect on brain, cognition, and neuropsychiatric disorders [16,17]. Various interventions [18,19], along with two recently published cohort studies [20,21], have found higher nut consumption was associated with lower depression scores. A cohort study in Tianjin, China indicated that nut consumption may be beneficial in the prevention of depressive symptoms [21]. In a large, parallel-group, multicenter, RCT using a Mediterranean diet supplemented with 30 g mixed nuts (15 g walnuts, 7.5 g hazelnuts, and 7.5 g almonds) including 7447 participants for 3 years, the result indicated that the inverse association with depression was not significant (RR = 0.78, 95% CI 0.55–1.10) in the whole cohort. However, when the analysis was restricted to participants with T2DM, a 40% lower risk of depression in participants with T2DM (RR = 0.59, 95% CI 0.36–0.98) was reported [18].

Almond is the most consumed and studied nut. It possesses potential prebiotic properties; its ingestion leads to an improvement in the gut microbiota profile and a modification of the gut microbiota activities [22].The study of Liu et al. [23] indicated that almond supplementation could significantly increase the populations of *Bifidobacterium* and *Lactobacillus*, which may potentially contribute to increased production of short-chain fatty acids(SCFAs) [24]. SCFAs are the ligand of G protein-coupled receptor43 (GPR43), and its combination with GPR43 can promote the secretion of glucagon-like peptide 1 (GLP-1) [25]. GLP-1 is an important regulator of microbiota–gut–brain signaling in health and stress-related psychiatric illnesses, which has been proven to have antidepressant effect [26].

In our previous preliminary study, we replaced 150 g/d staple food with 56 g/d almond to form an almond-based LCD (a-LCD) [15]. There are very few studies that have explored the role of a-LCD on both the depression and glycometabolism in T2DM patients. Based on the above, we hypothesized that a-LCD could improve depression and glycometabolism in patients with T2DM through modulating gut microbiota and GLP-1. In this study, we further explored the effect of a-LCD on the depression, HbA1c, gut microbiota and GLP-1.

## 2. Materials and Methods

### 2.1. Study Design

This was a 3-month, prospective RCT performed at a diabetes club and the Endocrine Division of the First and Second Affiliated Hospital of Soochow University from December 2018 to December 2019. The recruited participants were randomly allocated to either an intervention or control group using a table of computer-producing random numbers. The random numbers were concealed by someone who was not responsible for this study, so both the researcher and the participants were blinded prior to assignment. Before intervention, all participants underwent a one-week washout period to diminish the effect of background diets [27]. In this regard, participants who met the inclusion criteria were asked to stop eating nuts for >4 d/w before the intervention. This study followed the Declaration of Helsinki and the Guidelines for Good Clinical Practice and was approved by the ethics committee of the Soochow University (No. 2019000147). All enrolled patients signed consent form. The Randomized Controlled Trial Registration number for the study is ChiCTR1900024407.

### 2.2. Study Participants

Inclusion criteria of this study were as follows: patients (1) had been diagnosed with T2DM; (2) were older than 18 years; (3) had no change in oral anti-diabetic drugs or insulin in half a month before the intervention; (4) were able to communicate with others; (5) had volunteered to participate in this study, and (6) were able to provide informed consent. Patients were excluded from this study if they (1) ate nuts regularly (>4 days/week) [28]; (2) were allergic to nuts or received other dietary interventions; (3) had difficulty in chewing nuts; (4) had severe conditions including digestive dysfunction, heart failure, renal failure, malignant tumors, severe cerebrovascular diseases, ketosis, hyperthyroidism, liver dysfunction, or severe gallbladder and pancreatic diseases; (5) their FBG were more than 16.7 mmol/L [15];(7) suffered from mental illness; (8) used anti-depressant, anxiety, neurological or psychiatric medication [29]; (9) pregnant or lactating [29]; (10) used GLP-1 receptor agonists or Dipeptidyl Peptidase-4 (DPP-4) inhibitors;(11) regularly took glucocorticoids;(12) regularly took antibiotics, supplementations (e.g., prebiotics) and yogurt within 3 weeks which were known to alter gut microbiota [30]. Patients were withdrawn from the study if the following occurred: patients (1) ate almond <4 d/w (56 g/d) in the a-LCD group and ate almond in the LFD group >2d/w (56 g/d); (2) were unable to follow the diet plan; (3) suffered from major life events; (4) had adverse effect occurred during the intervention (e.g., intolerance to almonds).

### 2.3. Sample Size Calculation

Evidence from the literature showed that the mean difference of the changes in the depression score was 2 between the LCD and the LFD group [31]. Therefore, we calculated 23 participants for each group, with α = 0.05 and power = 0.80. Considering the 10% sample loss rate, the increase of sample size according to the actual situation is expected to be 25 cases in each group.

### 2.4. Intervention

The control group adopted an LFD education program which is a named six-point formula [15] developed by our research team based on the diabetes dietary guideline. The detailed contents of the six-point formula included: 1 jin vegetables/days; 2 liang staple food/meal (300 g/day); 3 tablespoons of oil/day; eating fruits under 4 conditions (stable glycemia, low glycemic index fruit, between two meals, adding energy fruit provided to the total calories); 5 kinds of protein food/day (1 carton of milk (220 mL), 1 egg, 1 liang fish or shrimp, 1 liang soybean production, 1 liang meat); 6 g salt/day. The a-LCD group consumed 56 g/day almond which replaced 150 g/d staple food (1 liang staple foods/meal) that is rich in carbohydrate, and the remaining of the dietary regimen kept the same as those of LFD. Almonds were uniformly purchased, weighed, vacuum-packed, and distributed every two weeks. The participants were instructed to consume almonds between meals or with breakfast, or when hungry. For those whose FBG were higher than normal (>6.1 mmol/L), the almonds were required to be consumed before 10:00 a.m. [32]. The participants were instructed to consume 50% of the nuts before bedtime if there was a risk of a nocturnal hypoglycemic event. Participants were followed up once a week, including where we: collected the information about the implementation of dietary regimen and almond adherence to understand the dietary adherence; strengthened their compliance and excluded participants with poor compliance; collected information of anti-diabetics; asked if they had any adverse reactions, hypoglycemic events, and major life events.

### 2.5. Outcomes

#### 2.5.1. Glycated Hemoglobin (HbA1c)

Blood samples were obtained to measure HbA1c at the nursing School of Soochow University and measured by high-performance liquid chromatography using Afinion AS100 Analyzer (Alere, Inc., Shanghai, China) in the molecular laboratory of the nursing school of Soochow University.

#### 2.5.2. Depression

Depression score was assessed through PROMIS short form v1.0-Depression 8b [33], which are universal rather than disease-specific. The PROMIS Depression item banks assess self-reported negative mood (sadness, guilt), views of self (self-criticism, worthlessness), and social cognition (loneliness, interpersonal alienation), as well as decreased positive affect and engagement (loss of interest, meaning, and purpose). Our team investigated local patients with T2DM and found that its cronbach’s α was 0.879. The total scores ranged from 37.1 to 81.1 points. The higher the score of the scale, the more serious the depressive symptoms.

#### 2.5.3. Anthropometric Measurements

The weight and height were measured bya unified measuring device at the nursing school of Soochow University.Body mass index (BMI) was calculated as weight (in kilograms) divided by height (in meterssquared).

#### 2.5.4. Dietary Record

Patients maintained a dietary record, including a detailed diet of one day of the weekend and two working days, and then *Feihua nutrition software V2.72 version* (Bowen Shixun Technology, Beijing, China) was used to calculate the proportion of three macro-nutrients per day to evaluate the implementation of the dietary plan of the subjects. An almond adherence record table was used to measure the compliance of almond intake, and consumption ≥4 days/week indicates good compliance.

#### 2.5.5. Fasting GLP-1 Concentration

GLP-1 was measured byenzyme-linked immunosorbent assay (ELISA). The fasting peripheral venous blood of participants was collected by vacuum blood vessels containing EDTA anticoagulants, DPP-4 inhibitors were added immediately after blood collection at bedside to prevent GLP-1 degradation. The blood samples were centrifuged at 2~8 °C 1000× *g* for 15 min within 30 min after collection and thesupernatant was retained and stored at −20 °C or −80 °C, but repeated freeze-thaw was avoided.

#### 2.5.6. Gut Microbiota

The fecal specimens of all participants were taken. Researchers instructed the participants to put the feces on the thick sterile pad. The sterile cotton swab was used to take about 20 g fresh fecesfrom participants into the sterile feces collection tubes. After the specimen was collected, it was immediately put into the portable liquid nitrogen tank and frozen for 15 min. The specimens were transferred to the −80 °C refrigerator within 24 h for preservation. For each fecal specimen, DNA was extracted and purified, and the V3-V4 region of the 16S rRNA genes was polymerase chain reaction (PCR)-amplified using modified universal bacterial primers [34]. Purified PCR products were sequenced with the Hiseq2500 PE250 platform [35]. Sequence data were compiled and processed using QIIME [36]. Sequence data were screened and filtered for quality and then aligned to the full-length 16S rRNA gene, using the SILVA reference alignment as a template [37]. Sequences were grouped into operational taxonomic units (OTUs) of 97% similarity. Phylogenetic classification was used to describe the intestinal composition of each participant [38]. The following alpha-diversity measures were assessed: (1) Chao1 index, a measure of species richness that is particularly applied to low abundance datasets; (2) Faith’s Phylogenetic Diversity (Faith’s PD), the most commonly used measure of phylogenetic diversity. The principal co-ordinates analysis (PCoA) of the weighted Unifrac (quantitative, i.e., weighs branches of phylogenetic tree based on abundance) distances was used to compare the overall microbiota structures in the two groups.

### 2.6. Statistical Analysis

A statistical analysis was performed using SPSS 25.0 software (SPSS, Inc., Chicago, IL, USA). For continuous variables, the results were described as the mean ± standard deviation (SD) and comparisons were performed using Independent Samples t-test, Paired Samples t-test or the Mann–Whitney U test, and Wilcoxon rank-sum test. For categorical variables, the results were presented as frequency (percentages); comparisons between groups were made using the Chi-squared test. To eliminate the problem of confounding factors, the comparisons of outcomes between the groups after the intervention were performed using a covariance analysis (covariate: age, protein, baseline level for depression and HbA1c, the rate of change of anti-diabetics).The intention-to-treat (ITT) of depression and HbA1c were performed so as to ensure the reliability of the research results. The proportion of three macro-nutrients in relation to energy supply, representing participants’ dietary adherence, which were assessed once a week during the intervention have been presented as a pie chart. The comparisons of gut microbiota between two groups were evaluated with theMann–Whitney U test, within group differences were analyzed using Wilcoxonrank test. A *p* value of <0.05 was considered statistically significant. A corrected false discovery rate (FDR) <0.2 was considered as significant [39].

## 3. Results

### 3.1. Study Participants

**Figure 1:**
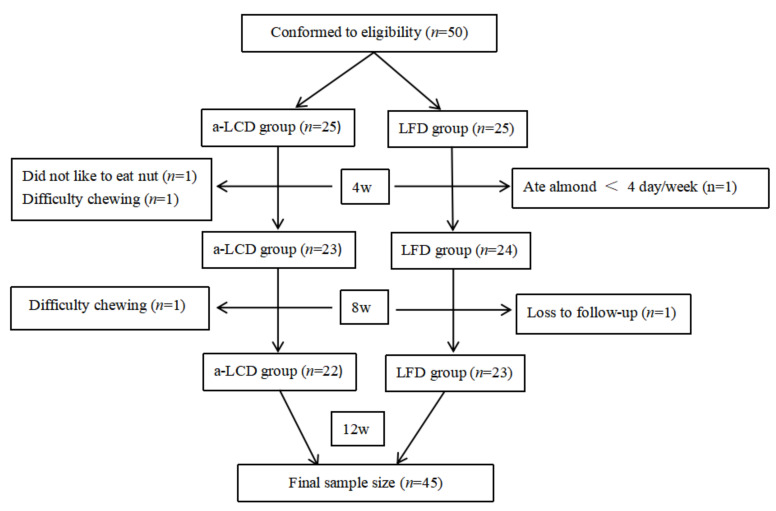
Flow diagram of the participants included in the study (a-LCD (almond-based low carbohydrate diet); LFD (low fat diet).

On the basis of inclusion and exclusion criteria, 50 participants with T2DM were recruited and randomly allocated to the a-LCD group (*n* =25) and the LFD group (*n* =25). Three participants (1 who did not like almond, 2 with difficulty chewing) in the a-LCD group and two participants (1 who ate almond <4 day/week, another who was lost during follow-up) in the LFD group withdrew from the study. Finally, the data of 22 participants in the a-LCD group and 23 in the LFD group were analyzed (Figure 1). The mean age of participants was (71.98 ± 5.63 years) and 25 (55.6%) were female. The general characteristics of the enrolled participants in each group are shown in Table 1. There were no statistically significant differences in any of the parameters between the two groups (*p* > 0.05).

### 3.2. Dietary Adherence

#### 3.2.1. Proportions of Calories from Three Macro-Nutrients the Patients Consumed

**Figure 2:**
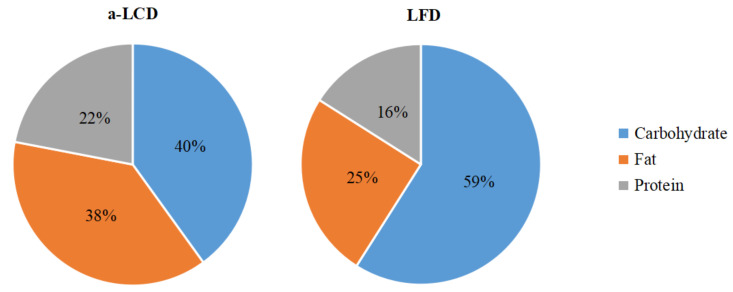
Three macro-nutrient energy supply ratios of two groups after intervention.

The results of the three macro-nutrients allocated between the a-LCD and the LFD group based on three days diet record showed that at the baseline, the total energy and the proportions of calories from the three macro-nutrients were not significantly different between the two groups. At the third month, the total energy was not significantly different between the two groups. However, compared to the LFD group, the calories from carbohydrates decreased, while those from fat significantly increased in the a-LCD group (*p* < 0.01) (Table 2). In addition, after the intervention, the percentage of the calories from carbohydrates (40%) met the standard of LCD in the a-LCD group, while the calories from fat (25%) met the standard of LFD in the LFD group (Figure 2).

#### 3.2.2. Almond Adherence

**Figure 3:**
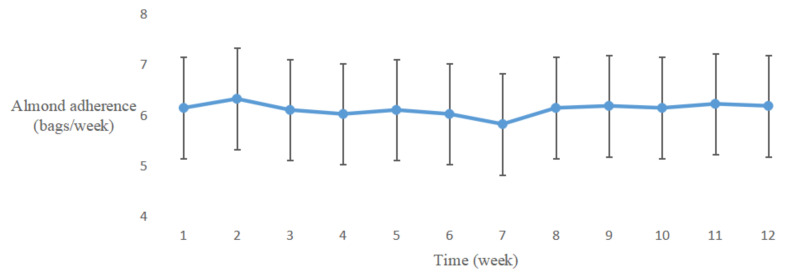
The changing trends of almond adherence in the a-LCD group.

The almond adherence of the a-LCD group is shown in Figure 3. A fold line diagram was performed to describe the changing trend of almond adherence in the a-LCD group. The result indicated that the frequency of almond consumption was stable and good.

### 3.3. Effect of a-LCD on Glycated Hemoglobin (HbA1c)

At the baseline, HbA1c levels were not significantly different between the two groups. Compared to the baseline, HbA1c levels in both groups decreased significantly (*p* < 0.01, *p* < 0.05) during the study period. At the third month, the HbA1c level in the a-LCD group decreased more than that in the LFD group (*p* < 0.01, Table 3). The ITT, in relation to HbA1c levels, was performed to ensure the stability of the above results. The result of ITT was found to be in agreement with the findings above (Supplementary Table S1).

### 3.4. Effect of a-LCD on the Changes of Anti-Diabetics

During the intervention, three in the LCD group and five in the LFD group had anti-diabetic dosage reduced, while the other participants remained unchanged, but there was no significant difference between the two groups in the third month (*p* > 0.05, Table 4)

### 3.5. Effect of a-LCD on Weight and BMI

At the third month, weight and BMI showed no significant difference between the two groups(*p* > 0.05, Table 5). Compared to the baseline, weight and BMI of a-LCD group improved significantly at the third month (*p* < 0.05, Table 5).

### 3.6. Effect of a-LCD on Depression

At baseline, the depression scores were not significantly different between the two groups. Compared to the baseline, depression score in the a-LCD group decreased significantly (*p* < 0.01) in the third month, while there was no decrease in the LFD group (*p* > 0.05). At the third month, the depression score in the a-LCD group was significantly lower than that in the LFD group (*p* < 0.01, Table 6). The ITT, in relation to depression scores, was performed to ensure the stability of the above results. The ITT result was found to be in agreement with the earlier findings (Supplementary Table S2).

### 3.7. Fasting Plasma GLP-1 Concentration

Compared to the baseline, we found that GLP-1 did not significantly change in both groups at the third month. However, GLP-1 in the a-LCD group was significantly higher than that in the LFD group after intervention (*p* < 0.05). (Table 7)

### 3.8. Gut Microbiota

A total of 2,981,481 quality reads of 90 samples were generated with an average of 33,128 ± 2868 reads per sample.

#### 3.8.1. Alpha-Adversity

The alpha-diversity of the gut microbiota community indicated that compared with the baseline, the Chao 1 and PD index of both groups increased significantly (*p*< 0.01) (Supplementary Figures S1 and S2).

#### 3.8.2. Beta-Diversity

The PCoA of the weighted Unifrac distances was used to compare the overall microbiota structures in the two groups and the results are shown in Supplementary Figures S3 and S4.The more similar the samples of community microbials are, the closer they are to each other.The result revealed there was no extremelyapparent separation in gut microbiota structure between and within the groups at the baseline and the third month.

#### 3.8.3. The Comparison of the Composition of Gut Microbiota in the Two Groups

**Figure 4:**
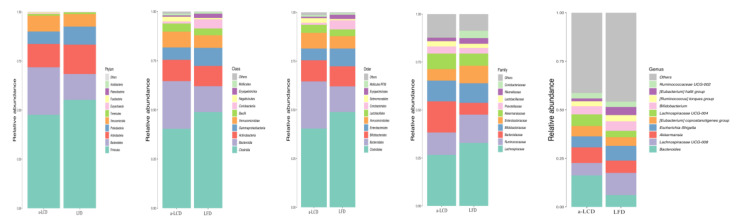
The comparison of the overall microbial composition between the two groups at the baseline. Only taxonomic groups 1% or greater are shown.

**Figure 5:**
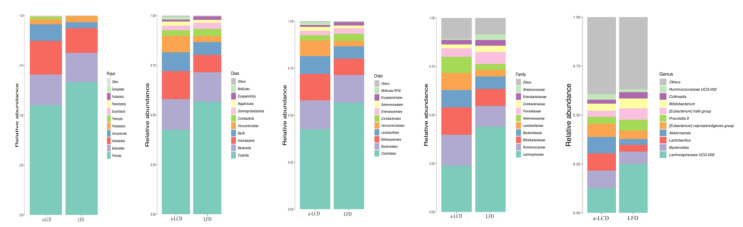
The comparison of the overall microbial composition between the two groups at the third month. Only taxonomic groups 1% or greater are shown.

The overall microbial compositions between two groups at baseline and three months are shown in the Figure 4 and Figure 5. To determine if the a-LCD affected the gut microbiota, we compared the composition of gut microbiota in the two groups at baseline and the third month (Table 8). At the phylum level, *Firmicutes* in the a-LCD group was significantly lower than that in the LFD group by the third month (*p* < 0.05, FDR = 0.026). Compared to the baseline, *Bacteroidetes* (*p* < 0.05, FDR = 0.016) decreased significantly in the a-LCD group. At the genus level, *Roseburia* (*p* < 0.01, FDR <0.01,) and *Ruminococcus* (*p* < 0.05, FDR = 0.073)in the a-LCD group were significantly higher than those in the LFD group by the third month; compared to the baseline: *Eubacterium*(*p* < 0.01, FDR = 0.013), *Roseburia* increased significantly(*p* < 0.05, FDR = 0.021) and *Bacteroides*(*p* < 0.05, FDR = 0.013)decreased significantly in the a-LCD group.

## 4. Discussion

Until now, only few studies have reported whether a diet regimen is beneficial to both depression and glycometabolism in T2DM patients. Almond is rich in some nutrients and it may be protective against the development of depression.Based on our previous study of a-LCD improving glucose [15], we further explored the effect of this diet regimen on both depression and glycometabolism in T2DM patients.

### 4.1. Effect of a-LCD on Glycemic Control and Anti-Diabetics

For diabetes patients with depression, the primary clinical concern is still on the control of glycometabolism. HbA1c level can reflect mean blood glucose level over the last 8–12 weeks and long-term glycemic control of T2DM patients [8]. It could be used as the main indicator to establish glycemic control. Over 3 months, our study found that both a-LCD (*p* < 0.01) and LFD (*p* < 0.05) improved HbA1c level, while better HbA1c level was seen in the a-LCD group(*p* < 0.01), which is consistent with our previous research [15].Although there was no significant difference inanti-diabetics between the two groups during the three-month period, three in the a-LCD group and five in the LFD groupreduced the dosage of anti-diabetic drugs. After controlling the rate of change of anti-diabetics as a covariate, the HbA1c in the a-LCD group was still significantly lower than that in the LFD group, indicating that the hypoglycemic effect of LCD was better than that in the LFD group. The reasons for the potential benefit of a-LCD on HbA1c were shown in those studies [15,27].

### 4.2. Effect of a-LCD on Weight and BMI

Nuts have rich energy density and high fat [40], the greater fat availability may reduce gastric emptying rate to increase the satiety [41]. In this study, a-LCD decreased weight (*p* < 0.05) and BMI (*p* < 0.05) of patients with T2DM, further confirming the effectiveness of LCD in improving weight and BMI. The results are in agreement with the result of our previous study and the reason for the potential benefit of a-LCD on weight and BMI were shown in that study [15].

### 4.3. Effect of a-LCD on Depression Score

The impact of LCD on depression is controversial. Brinkworth et al. [42] used monounsaturated and polyunsaturated fat instead of carbohydrate to form LCD and assessed the effect of LCD on psychological mood state and well-being in obese patients with T2DM. The results found that the Beck Depression Inventory Score declined over time in theboth LCD and LFD groups (*p* < 0.05). Saslow et al. [43] assessed the effect of a 3-month LCD (lower their carbohydrate consumption and derive their remaining calories from fat) and a LFD on psychological outcomes in obesity/overweight patients with T2DM or pre-diabetes. No significant differences in depression scores were observed between the two groups (*p* > 0.05). In the current study, we found that a-LCD significantly decreased the depression score compared to LFD (*p* < 0.01) in patients with T2DM, which is not consistent with the findings in above-mentioned studies. The important reason for the inconsistent effect of LCD on depression may be due to the difference in the composition of the food for LCD. A major difference was that we used almond to replace carbohydrate. Almond as a commonly eaten nut, is a rich source of dietary fiber, polyphenols, and unsaturated fatty acids [44], that may be protective against the development of depression [45,46,47]. With regard to the association of nuts intake and mental health, most of studies showed the positive effect of nuts. In the PREDIMED trial, an RCT with the Mediterranean diet supplemented with nuts showed a 40% lower risk of depression in patients with T2DM (RR = 0.59, 95% CI 0.36–0.98) [18]. An RCT conducted by Pribis et al. [19] explored the effect of walnut consumption on mood in young adults and observed a significant medium effect size improvement in the Total Mood Disturbance Score (−27.5%, *p* = 0.043) in males. Another reason for the inconsistent results with other studies may be the different participants in their studies who were obese.

### 4.4. Effect of a-LCD on Regulation of Gut Microbiota and GLP-1 Expression

In our study, the a-LCD and LFD at 3 months increased Chao1 and PD index in the two groups, although there were no significantdifferences between the two groups. Increased microbiome diversity could promote greater stability of the microbiome in the long term, thereby contributing to functional resilience against extreme stress and perturbations [48].

Currently, there are no reports of gut microbiota in T2DM with depression, but there is evidence of significant differences between depressed patients and healthy people [49]. Jiang et al. [49] compared the composition between depressed patients with healthy people, and the result indicated that, at the phylum level, depressed patients had higher population of *Bacteroidetes*. After the intervention of the a-LCD, we observed the changes of the above-mentioned bacteria. At the phylum levels, *Bacteroidetes* decreased significantly in the a-LCD group. We also found that a-LCD for 3 months decreased the population of the pathogenic bacterium *Bacteroides*, which is consistent with the study of Dhillon et al. [50]. The above result show that a-LCD could regulate the gut bacteria and the improvement of depression score in the a-LCD may be through modulating of the microbiome-gut-brain.

A growing understanding of the microbiome-gut-brain connections is revealing the pathways by which food choices may affect depression [51], and GLP-1 is an important regulator of microbiota-gut-brain signaling in health and stress-related psychiatric illnesses [26]. Almonds are a rich source of fiber, unsaturated fats, and polyphenols, all nutrients that can favorably alter the gut microbiome [50] and its consumption increases the number of SCFAs-producing bacteria [23]. SCFAs in combination with GPR43 can promote the secretion of GLP-1 [25]. GLP-1, produced both in peripheral L-cells of the gastrointestinal tract and in the brain, is an anorexigenic peptide and treatment target for T2DM [26]. GLP-1 analog liraglutide has been recently reported to attenuate depressive behaviors via improving hippocampal plasticity in mice [52]. GLP-1 receptor agonists have also been shown to have neuroprotective effects both in diabetic [53] and depressed [54] mice. The study of Grant et al. [55] showed that a depression and anxiety score generally tends to improve in GLP-1 analog exenatide-treated T2DM patients and it could be used as an adjunctive therapy for depression in diabetes.

An important finding of the present study was that the a-LCD significantly increased the relative abundance of SCFAs-producing bacteria*Roseburis* [24] (*p* < 0.01, FDR < 0.01) and *Ruminococcus* [24](*p* < 0.05, FDR = 0.073), which is consistent with the study by Holscher et al. [56], where they found that almond consumption increased the *Roseburia* (*p* < 0.05). Compared to the baseline, SCFAs-producing bacteria *Roseburia* (*p* < 0.05, FDR = 0.021) and *Eubacterium* (*p* < 0.01, FDR = 0.013) were significantly increased in the a-LCD group. However, other studies found that almond consumption also increased the population of SCFAs-producing bacteria *Bifidobacterium* and *Lactobacillus* [23], although these were not consistent with our findings, which may be due to the differences in ethnicity [57].

Research has indicated that GLP-1 levels decrease significantly in the T2DM patients [58]. In our study, GLP-1 concentration decreased at the third month compared to the level of the baseline, although there were no statistical differences. However, it is worth noting that GLP-1 concentration in the LCD group was significantly higher than that in the LFD group in the third month (*p* < 0.05), indicating a-LCD maintained the GLP-1 secretion. An RCT randomly assigned a trial diet with 28 g almonds or without almonds and the result indicated that the rise in GLP-1 concentration at 30 min post-meal was due to the effect of almond ingestion for both non-diabetic and T2DM groups [59].Based on the results of this study, we speculate that the role of a-LCD in improving depression in patients with T2DM may be associated with it stimulating the growth of SCFAs-producing bacteria, then increasing SCFAs and GPR43 activation, further maintaining GLP-1 secretion. However, we did not observe the change in SCFAs and GPR43 activation due to the limited fund. In further study, the SCFAs and GPR43 activation should be explored.

## 5. Conclusions

Based on the findings of this study, the a-LCD may be an effective intervention for the improvement of both depression and glycometabolism in patients with T2MD. A-LCD significantly increased the SCFAs-producing bacteria and maintained the GLP-1 secretion. We speculate that a-LCD improving depression in T2DM patients may be associated with it stimulating the growth of SCFAs-producing bacteria, increasing SCFAs production and GPR43 activation, and further maintaining GLP-1 secretion. In futurestudies, the SCFAs and GPR43 activation should be further examined.

## 6. Limitations

There are several limitations of our study. One of the limitations is that the present study was conducted in a T2DM population with and without depression, which may limit the a-LCDeffect on depression. Further studies that examine the effects of a-LCD in patients with diabetes with clinical depression are required to understand the effectiveness of the current findings. We did not monitor the dynamic changes of depression over the three-month period; therefore, we could not find the earliest time point for improvement in depression. Due to the limitation of fund, the analysis method of gut microbiota needs to be improved in the further study.

This will be taken into consideration in future research.

## Figures and Tables

**Table 1:** Baseline characteristic.

Variables		a-LCD (*n* = 22)	LFD (*n* = 23)	t/χ^2^/Z	*p*
		x ± SD/n(%)/ M (P25,P50)	x ± SD/n(%)/ M (P25,P50)		
**Demographic data**					
Age (years)		73.55 ± 4.99	70.48 ± 5.91	−1.877 ^a^	0.067
Gender-male		9 (40.9%)	11 (47.8%)	0.218 ^b^	0.641
Marital status (married)		20 (90.9%)	21 (91.3%)	0.002 ^c^	0.963
Education	Primary and below	2 (9.1%)	2 (8.7%)	4.037 ^b^	0.258
	Journal high school	4 (18.2%)	10 (43.5%)		
	Technical and senior high school	11 (50.0%)	9 (39.1%)		
	Journal college school and above	5 (22.7%)	2 (8.7%)		
Payment	Medical insurance	22 (100%)	21 (91.3%)	2.002 ^c^	0.157
Monthly income (thousand yuan)	<2	0 (0%)	3 (13.0%)	3.950 ^b^	0.139
	2~5	19 (86.4%)	15 (65.2%)		
	≥5	3 (13.6%)	5 (21.7%)		
Occupation status	Retire	22 (100%)	22 (95.7%)	0.978 ^c^	0.323
	On the job	0 (0%)	1 (4.3%)		
Residential status	Living by oneself	2 (9%)	2 (8%)	0.311 ^b^	0.856
	Living with spouse	19 (86%)	19 (83%)		
	Living with children	1 (5%)	2 (9%)		
Exercise intensity (d)	Low intensity	19 (86.4%)	18 (78.3%)	0.505 ^b^	0.477
	Moderate intensity	3 (13.6%)	5 (21.7%)		
Exercise time/(minute)		315.00(210.00,420.00)	360.00(210.00,420.00)	−0.537 ^d^	0.591
**Clinical data**					
Smoking (Yes)		1 (4.5%)	2 (8.7%)	0.311 ^c^	0.577
Drinking (Yes)		4 (18.2%)	3 (13.0%)	0.226 ^c^	0.634
Diabetes duration (years)		14.18 ± 7.06	15.65 ± 7.02	0.700 ^a^	0.487
Family history of diabetes (Yes)		12 (54.5%)	11 (47.8%)	0.203 ^b^	0.652
Diabetic complication (Yes)		9 (40.9%)	11 (59.3%)	0.573 ^b^	0.449
Accompanying disease (Yes)		16 (72.7%)	17 (73.9%)	0.008 ^b^	0.928
Therapy method	None	1 (4.5%)	1 (4.3%)	1.825 ^b^	0.610
	Only Hypoglycemic drugs	12 (54.5%)	14 (60.9%)		
	Only Insulin	1 (4.5%)	3 (13.0%)		
	Hypoglycemic drugs + insulin	8 (36.4%)	5 (21.7%)		
Number of combination medication	0	2 (8.7%)	4 (14.8%)	5.022 ^b^	0.170
	1	8 (34.8%)	4 (14.8%)		
	2	13 (56.5%)	16 (59.3%)		
	3	0 (0%)	3 (11.1%)		

a-LCD (almond-based low carbohydrate diet); LFD (low fat diet); ^a^ Independent-samples T test; ^b^ Pearson chi-square; ^c^ Yates’ correction chi-square; ^d^ Mann–Whitney U. M(P25,P50): median (25th and 75th percentile).

**Table 2:** Comparison of the calories from three macro-nutrients consumed by the patients.

	Variables	a-LCD (*n* =22)	LFD (*n* =23)	t	*p*
Baseline	Total calorie intake/day	1686.34 ± 231.25	1781.91 ± 280.91	−1.232	0.184
	Carbohydrate-calorie (Kcal)	974.95 ± 148.22	1007.61 ± 136.32	−0.761	0.504
	Fat-calorie (Kcal)	406.76 ± 143.88	478.29 ± 149.97	−1.614	0.085
	Protein-calorie (Kcal)	318.45 ± 63.19	292.38 ± 65.12	1.348	0.524
Third month	Total calorie intake/day	1642.08 ± 227.74	1764.77 ± 297.40	−1.536	0.114
	Carbohydrate-calorie (Kcal)	673.14 ± 91.80	1042.10 ± 195.41	−8.016	<0.01 **
	Fat-calorie (Kcal)	648.19 ± 128.93	433.01 ± 137.39	5.357	<0.01 **
	Protein-calorie (Kcal)	372.03 ± 64.45	288.94 ± 64.34	1.962	0.067

*p* Value for comparison by independent sample t-test. **: *p* < 0.01.

**Table 3:** Comparison of glycated hemoglobin (%) between the two groups.

Study Period	a-LCD (*n* =22)	LFD (*n* =23)	t/F	*p*
Baseline	7.67 ± 1.60	7.54 ± 1.31	−0.287 ^a^	0.776
Third month	6.85 ± 1.02 (adjusted:6.77 ± 0.13)	7.37 ± 1.29 (adjusted:7.44 ± 0.12)	14.111 ^b^	<0.01 **
t	4.081 ^c^	2.614 ^c^		
*p*	<0.01 **	0.016 *		

^a^ Independent-samples T test for between-groupdifferencesat the baseline; ^b^ covariance analysis for between-groupdifferences at the third month, with adjusted data presented as mean ± standard error (covariate: age, baseline HbA1c, protein, the rate of change of anti-diabetics); ^c^ paired sample T test for within-group difference. * *p* < 0.05; ** *p* < 0.01.

**Table 4:** Comparison of other anti-diabetic drugs between the two groups.

	a-LCD (*n* = 22)	LFD (*n* = 23)	χ^2^	*p*
Reduction	3 (14%)	5 (22%)	0.019	0.889
No change	19 (86%)	18 (78%)		

*p* value for comparison between treatments diets by Chi-square test.

**Table 5:** Comparison of weight and BMI between the two groups.

Variables		a-LCD (*n* = 22)	LFD (*n* = 23)	t	*p*
Weight (Kg)	Baseline	66.60 ± 8.81	63.07 ± 12.88	0.784 ^a^	0.459
	Third month	59.34 ± 8.90	62.58 ± 13.12	0.967 ^a^	0.339
	t	2.164 ^b^	1.397^b^		
	*p*	0.042 *	0.176		
BMI (Kg/m^2^)	Baseline	23.53 ± 2.33	23.69 ± 2.83	0.216	0.830
	Third month	23.02 ± 2.45	23.53 ± 3.04	0.641	0.524
	t	−2.261	−1.283		
	*p*	0.034 *	0.211		

*p* value for comparison between two groups by Independent Samples t-test and paired sample T test. ^a^ Independent-samples T test for between-group differencesat the baseline and third month; ^b^ paired sample T test for within-group difference. * *p* < 0.05.

**Table 6:** Comparison of depression scores between the two groups.

Study Period	a-LCD (*n* = 22)	LFD (*n* = 23)	t/F	*p*
Baseline	48.41 ± 8.05	49.57 ± 8.46	0.471 ^a^	0.640
Third month	42.07 ± 5.80(adjusted:42.58 ± 0.89)	48.65 ± 7.69(adjusted:48.16 ± 0.87)	19.308 ^b^	<0.01 **
t	6.196 ^c^	0.838 ^c^		
*p*	<0.01 **	0.411		

^a^ Independent-samples T test for between-group differencesat the baseline; ^b^ covariance analysis for between-group differences at the third month, with adjusted data presented as mean ± standard error (covariate: age, baseline depression scores, protein); ^c^ paired sample T test for within-group difference. * *p* < 0.01.

**Table 7:** Comparison of GLP-1[M(P25,P75)], (pmol/L)] in the two groups.

Study Period	a-LCD (*n* =22)	LFD (*n* =23)	Z	*p*
Baseline	1.381 (0.697,3.157)	1.190 (0.804,1.896)	−0.409	0.683
Third month	1.092 (0.886,2.671)	0.630 (0.261,1.997)	−2.396	0.017 *
Z	−0.221	−1.339		
*p*	0.833	0.162		

Z-value and p-value for comparisons by Mann–Whitney U or Wilcoxon for between or within group differences at the baseline and third month, the result presented as M(P25, P75). * *p* < 0.05; M(P25,P50): median (25th and 75th percentile).

**Table 8:** Comparison of the composition of gut microbiota [M(P25,P75)] in the two groups.

Phylum	Genus	Study Period	a-LCD (*n* =22)	LFD (*n* =23)	Z	*p*(adj. val.)
*Firmicutes*		Baseline	0.389 (0.283,0.729)	0.544 (0.455,0.671)	−1.317	0.188 (0.194)
		Third month	0.580 (0.371,0.672)	0.684 (0.561,0.778)	−2.317	0.021 * (0.026)
		Z	−1.282	−2.281		
		*p*(adj. val.)	0.200 (0.213)	0.023 * (0.038)		
	*Roseburia*	Baseline	0.002 (0.000,0.005)	0.009 (0.005,0.024)	−1.892	<0.01 ** (<0.01)
		Third month	0.005 (0.000,0.006)	0.000 (0.000,0.001)	−2.626	<0.01 ** (<0.01)
		Z	−2.193	−4.075		
		*p*(adj. val.)	0.028 * (0.021)	<0.01 ** (<0.01)		
	*Eubacterium*	Baseline	0.008 (0.004,0.0220)	0.037 (0.018,0.070)	−3.747	<0.01 ** (<0.01)
		Third month	0.026 (0.004,0.057)	0.042 (0.024,0.099)	−2.082	0.037 * (0.073)
		Z	−2.678	−1.734		
		*p*(adj. val.)	<0.01 ** (0.013)	0.083 (0.052)		
	*Ruminococcus*	Baseline	0.017 (0.011,0.033)	0.020 (0.005,0.037)	−0.829	0.470 (0.407)
		Third month	0.026 (0.005,0.044)	0.005 (0.000,0.019)	−2.015	0.044 * (0.073)
		Z	−0.341	−2.312		
		*p*	0.733 (0.308)	0.021 * (0.020)		
	*Lactobacillus*	Baseline	0.007 (0.003,0.049)	0.005 (0.002,0.012)	−1.420	0.156 (0.223)
		Third month	0.007 (0.000,0.068)	0.000 (0.000,0.047)	−1.666	0.096 (0.245)
		Z	−1.150	−0.973		
		*p*(adj. val.)	0.130 (0.073)	0.330 (0.167)		
*Bacteroidetes*		Baseline	0.249 (0.120,0.323)	0.110 (0.072,0.180)	−2.793	<0.01 ** (0.011)
		Third month	0.151 (0.061,0.256)	0.108 (0.042,0.236)	−0.591	0.555 (0.415)
		Z	−2.451	−1.004		
		*p*(adj. val.)	0.014 * (0.016)	0.361 (0.188)		
	*Bacteroides*	Baseline	0.144 (0.057,0.256)	0.047 (0.023,0.119)	−3.244	<0.01 ** (<0.01)
		Third month	0.064 (0.027,0.106)	0.057 (0.009,0.085)	−0.978	0.328 (0.364)
		Z	−2.354	−0.335		
		*p*(adj. val.)	0.019 * (0.013)	0.735 (0.308)		

Z-value and *p*-value for comparisons by Mann–Whitney U or Wilcoxon for between or within group differences at the baseline and third month, the result presented as M (P25,P75). * *p* < 0.05; ** *p* < 0.01; M(P25,P50): median (25th and 75th percentile). *P* value (adj. val.), FDR < 0.2 was considered as significant.

## Supplementary Materials

The following are available online at https://www.mdpi.com/2072-6643/12/10/3036/s1, Supplementary Table S1: ITT of glycated hemoglobin (%) between the two groups; Supplementary Table S2: ITT of depression scores between the two groups; Supplementary Figure S1: The Comparison of Chao 1 and PD between the two groups (NS: no significant difference); Supplementary Figure S2: The Comparison of Chao 1 and PD within the groups (** *p*< 0.05; *** *p*< 0.01); Supplementary Figure S3: Comparison of beta-diversity of gut microbiota between two groups (A: Baseline; B: Third month); Supplementary Figure S4: Comparison of BetFa-diversity of gut microbiota within the group (A: a-LCD group; B: LFD group). The abscissa represents the first principal component, and the percentage represents the contribution value of the first principal component to the sample difference; the ordinate represents the second principal component, and the percentage represents the contribution value of the second principal component to the sample difference. Each point in the graph represents a sample, and the samples of the same group are represented by the same color.
