# Supplementary material for: An Almond-Based Low Carbohydrate Diet Improves Depression and Glycometabolism in Patients with Type 2 Diabetes through Modulating Gut Microbiota and GLP-1: A Randomized Controlled Trial
Source: Nutrients. 2020 Oct 3;12(10):3036. doi: 10.3390/nu12103036 (PMC7601479; doi:10.3390/nu12103036)
Supplement: Supplementary file 1 [file nutrients-12-03036-s001.pdf]

Supplementary Table S1. ITT of glycated hemoglobin (%) between the two groups.

Study period	a-LCD (*n*=25)	LFD (*n*=25)	t/F	*p*
Baseline	7.64±1.50	7.54±1.25	-0.246[a]	0.807
Third month	6.91±1.00(adjusted:6.88±0.12)	7.38±1.24(adjusted:7.42±0.12)	9.997[b]	＜0.01**
t	3.911[c]	2.587[c]		
p	＜0.01**	0.016*		

[a] Independent-samples T test for between-group differences at the baseline; [b] covariance analysis for between-group differences at the third month, with adjusted data presented as mean±standard error (covariate: age, baseline HbA1c, protein, the rate of change of anti-diabetics); [c] paired sample T test for within-group difference. * $p < 0.05$; * *$p < 0.01$; ITT: intention-to-treat.c

Supplementary Table S2.ITT of depression scores between the two groups .

Study period	a-LCD (*n*=25)	LFD (*n*=25)	t/F	*p*
Baseline	48.38±7.53	49.59±8.10	0.544[a]	0.589
Third month	42.81±5.79(adjusted:43.22±0.89)	48.74±7.37(adjusted:48.33±0.89)	15.596[b]	＜0.01**
t	5.627[c]	0.839[c]		
p	＜0.01**	0.410		

[a] Independent-samples T test for between-group differences at the baseline; [b] covariance analysis for between-group differences at the third month, with adjusted data presented as mean±standard error (covariate: age, baseline depression scores, protein); [c] paired sample T test for within-group difference. * $p < 0.05$; * * $p < 0.01$; ITT: intention-to-treat.

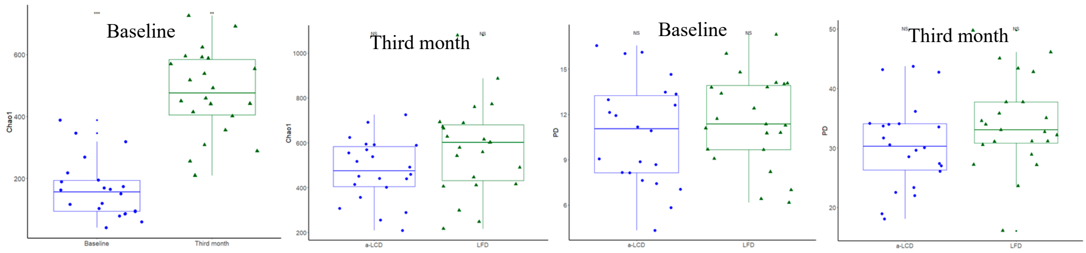

Supplementary Figures S1. The Comparison of Chao 1 and PD between the two groups (NS: no significant difference)

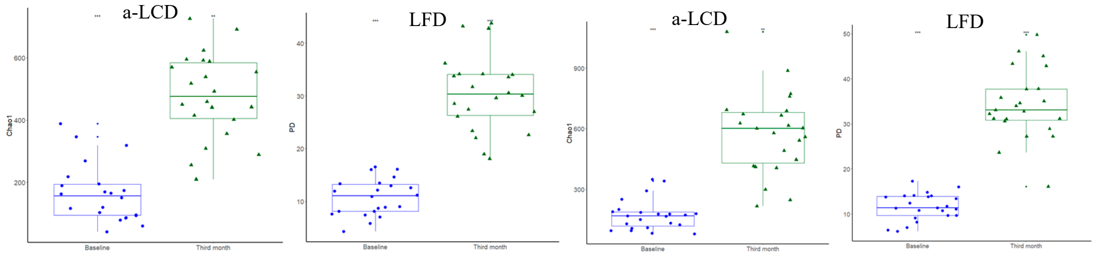

Supplementary Figures S2. The Comparison of Chao 1 and PD within the groups (** p< 0.05; * ** p< 0.01)

B

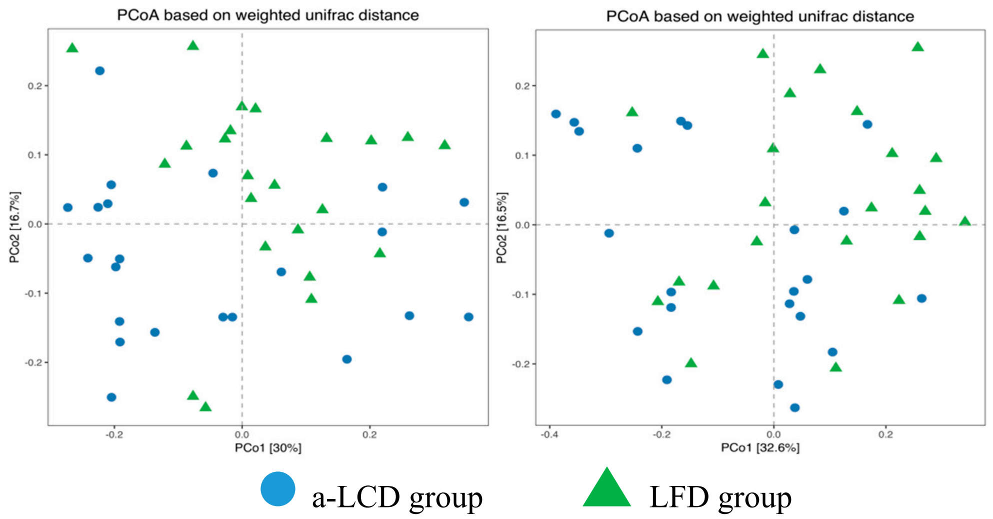

Supplementary Figure S3. Comparison of beta-diversity of gut microbiota between two groups (A:Baseline; B:Third month).

B

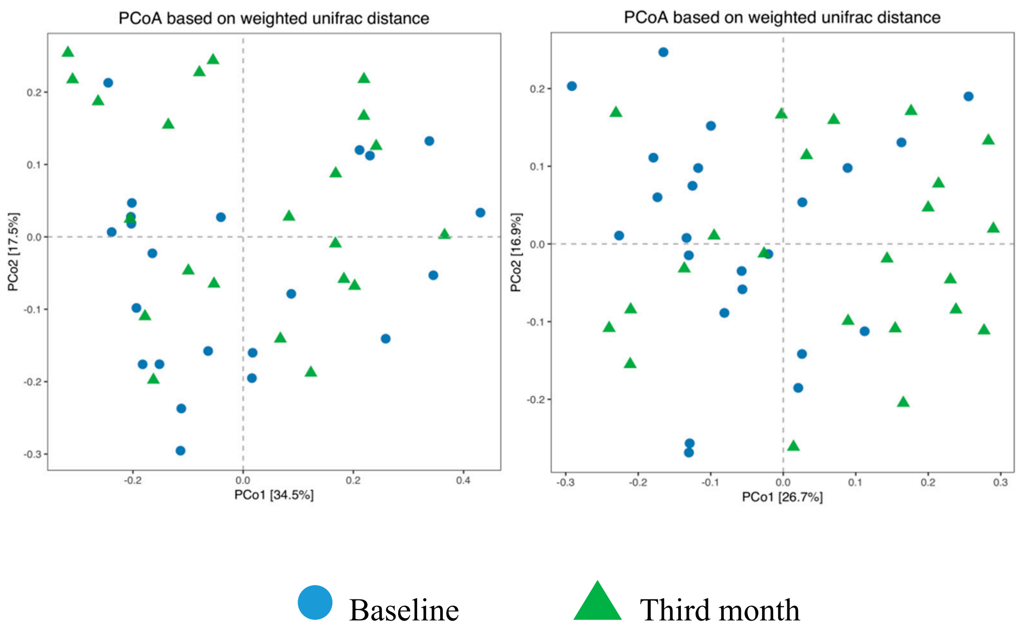

Supplementary Figure S4. Comparison of Beta-diversity of gut microbiota within the group (A: a-LCD group; B: LFD group). The abscissa represents the first principal component, and the percentage represents the contribution value of the first principal component to the sample difference; the ordinate represents the second principal component, and the percentage represents the contribution value of the second principal component to the sample difference. Each point in the graph represents a sample, and the samples of the same group are represented by the same color.
